# Leishmaniasis in Ethiopia: A systematic review and meta-analysis of prevalence in animals and humans

**DOI:** 10.1016/j.heliyon.2018.e00723

**Published:** 2018-08-07

**Authors:** Ayalew Assefa

**Affiliations:** Sekota Dryland Agricultural Research Center, P.O. Box 62, Sekota, Ethiopia

**Keywords:** Infectious disease, Epidemiology

## Abstract

Leishmaniasis is a neglected tropical disease caused by obligate intracellular protozoa of the genus *Leishmania*. Ethiopia does not have an overall estimation of prevalence of leishmaniasis infection at a country level. The objective of this systematic review and meta-analysis was to summarize and pool estimates of studies that report the prevalence of leishmaniasis in Ethiopia. The literature search was conducted to identify all published studies reporting the prevalence of leishmaniasis with clearly designed inclusion and exclusion criteria. From all screened articles, 30 studies were eligible for final meta-analysis and systematic review. Because substantial heterogeneity was expected, random-effects meta-analyses were carried out using the total sample size and number of positives to estimate the prevalence of the disease at a country level. Between-study variability was high (τ^2^ = 0.02; heterogeneity I^2^ = 99.72% with Heterogeneity chi-square = 11985.41, a degree of freedom = 33 and P = 0.001). The overall random pooled prevalence of leishmaniasis was 19% (95% CI 14%–24%). Meta-regression analysis showed that diagnosis method used have contributed to the heterogeneity of studies. Molecular diagnosis has significantly lower prevalence than microscopic examination with a coefficient of −0.32, a p-value of 0.024, and CI **(−**0.6–0.05). The result of effect estimates against its standard error showed there was no publication bias with a P value of 0.084. This review indicated that there is still a higher prevalence of Leishmaniasis in the country. Reporting on risk factors like sex and age affected, species of *Leishmania* involved and many more other risk factors reviewing was not possible in this study due to lack of completeness in articles included. However, this report is an indication that the country needs nationally coordinated extensive prevention and control plan to reduce public health and socio-economic impact of the disease.

## 1 Introduction

Leishmaniasis is a neglected tropical disease caused by obligate intracellular protozoa of the genus *Leishmania*. It is endemic in 88 countries in which 350 million people are at risk of infection. The impact of the disease is mainly on the impoverished population globally (Ngure et al., 2009). The increase in Leishmaniasis prevalence can be due to several reasons. Environmental conditions, socio-economic status, demographic and human behaviors pose significant risks for human Leishmaniasis (Oryan and Akbari, 2016). Poor housing system, economically forced migration to endemic foci of infection in search of work, suitable environmental habitat for vectors due to urbanization and deforestation, immunosuppression, and lack of personal protective measures are some of the risk factors implicated (Alvar et al., 2006). These environmental changes as well as population movements, probably alter the dynamics of vectors and reservoirs that may increase human exposure to the diseases (Oryan and Akbari, 2016).

Leishmaniasis is endemic to Ethiopia. Both Cutaneous (CL) and Visceral Leishmaniasis (VL) are growing health problems of the country (Ayele and Ali, 1984; Lemma et al., 1969). Serologically positive (asymptomatically) and confirmed cases of Visceral leishmaniasis have been reported from 8 regions (Tigray, Amhara, Oromia, Southern Nations and Nationalities People's Regional State, Somali, Afar, Gambella and Benshangul Gumuz regional states). The disease is well established in Metema and Humera plains in northern Ethiopia, the Omo plains, and the Aba Roba focus and Weyto River Valley in South Nations and Nationalities region. It was also reported from the Moyale area and Genale river basin in the Oromia regional state, Afder, and Liban zones in Ethiopia's Somali region, and the Awash Valley in the Afar regional state (Leta et al., 2014). Currently, more than 4,500 cases yearly in endemic areas have been reported. In the north, the vector is associated with *Red acacia* and Balanites trees, while in the south it is associated with termite hills (Abera et al., 2016; Hailu et al., 2009).

On the other hand, Cutaneous Leishmaniasis has been well known since 1913 and is endemic in most regions (Lemma et al., 1969) however, it is one of the neglected diseases in the country. There are an estimated 50,000 cases yearly with three clinical forms: localized CL, mucosal Leishmaniasis and diffuse cutaneous Leishmaniasis (DCL), all mainly caused by *Leishmania aethiopica* (Dassoni et al., 2017; Padovese et al., 2009), which are mainly found in the highlands of the country. The reservoir host is the rock hyrax while the vectors include *Phlebotomus longipes* and *Phlebotomus pedifer* sand flies (Ngure et al., 2009).

Despite a few reports on reservoirs hosts, the status of leishmaniasis in both domestic and wild animals in Ethiopia is not well studied. Planning effective control measures may require identifying, animal reservoir hosts (Hailu, 2016). According to some reports, the domestic dog, rodents, and carnivores are the reservoir host for VL in East Africa (Kalayou et al., 2011; Kassahun et al., 2015a; Kenubih et al., 2015). Many vector sand flies have also been implicated in transmitting the diseases in the country. The environmental conditions that favor the vector are very suitable in some parts of Ethiopia (Gebre-Michael et al., 2007; Hailu et al., 1995). Determining, the composition and distribution patterns of sand flies can indicate the possible presence of autochthonous transmission and/or can aid in the incrimination of the vector species (Yared et al., 2017).

Ethiopia does not have an overall estimation of prevalence of leishmaniasis at a country level. Meanwhile, there has been an increase in the number of studies on leishmaniasis prevalence across Ethiopia, especially in a human population. In the present systematic review and meta-analysis, we retrieved and reviewed publications relating to the disease in the country. The objective of this systematic review and meta-analysis (SR&MA) was to summarize and pool estimates that report the prevalence of leishmaniasis at the national or regional level. This study may help health policymakers to develop a better control and prevention programs that can minimize the devastating effect of the diseases on the livelihood of the poor.

## 2 Methodology

### 2.1 Literature search strategy

**Fig. 1:**
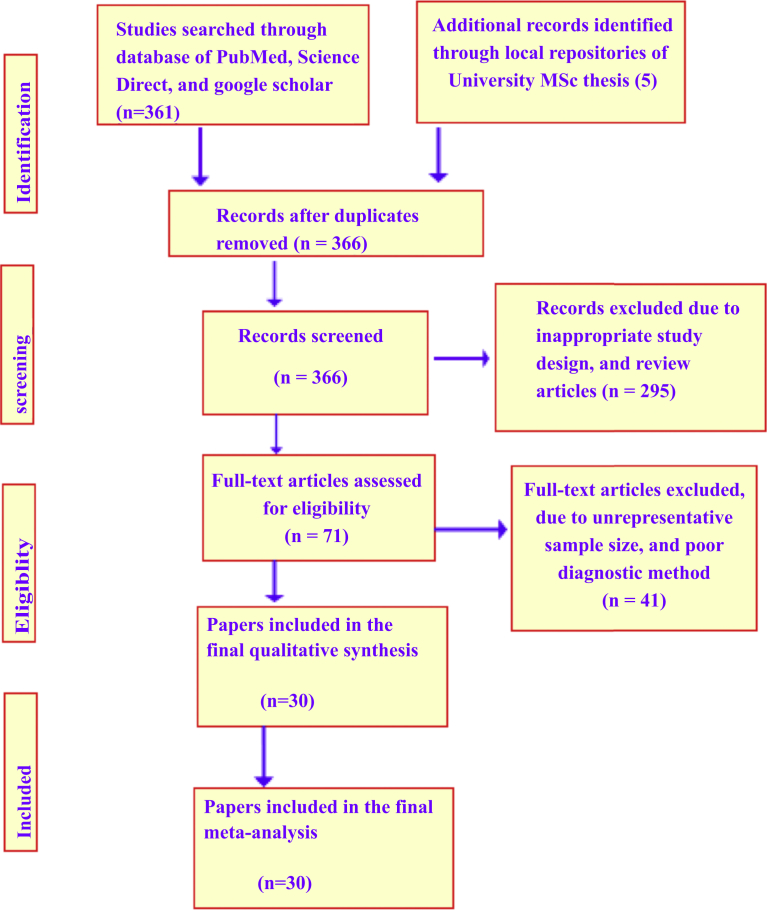
Flowchart of literature search and inclusion/exclusion process.

The literature search was conducted to identify all published studies reporting the prevalence and/or incidence of visceral and cutaneous leishmaniasis in animals and humans. The search was conducted in electronic databases of PubMed, CabDirect, Google scholar, African Journals Online, and Science Direct from August 2017 to April 2018. The specific search medical subject headings (MeSH) terms include “leishmaniasis and Ethiopia,” “leishmaniasis prevalence and Ethiopia,” “cutaneous leishmaniasis and Ethiopia,” “visceral leishmaniasis and Ethiopia,” “leishmaniasis in animals in Ethiopia,” “visceral leishmaniasis in animals in Ethiopia.” Based on the intensive literature search, a total of 366 pieces of literature that report on leishmaniasis in Ethiopia were retrieved. However, due to many eligibility related reasons, only 30 reports on the prevalence of leishmaniasis in animal reservoirs (5 studies) and humans (25 studies) made it to the final meta-analysis procedure. Study screening strategy and exclusion reasons are presented in Fig. 1.

### 2.2 Eligibility criteria and data extraction procedure

All Articles on leishmaniasis in Ethiopia were downloaded and added to Mendeley reference manager. Duplicates were rigorously checked and removed. Quality criteria were developed before starting the review of full papers. Inclusion/exclusion criteria were defined regarding the relevance of the articles to the research questions of interest. The inclusion criteria include an observational study that reports the prevalence of the diseases, published article or MSc thesis in university online repositories, reporting the prevalence of the disease from 1969 to 2017, diagnostic methods that employed one of the diagnostic approaches microscopic examination, culture, serological examination, and molecular diagnostic methods. Articles that met the above criteria were included. Publications that were poor in design (unrepresentative sample size, wrong sampling approach, fail to employ an appropriate diagnostic method, institution-based retrospective data reports without sound diagnostic approaches), despite reporting the prevalence of the disease, they were removed. Titles were checked twice in both excluded and included databases of the Mendeley reference manager before the start of the data extraction process. Those titles considered relevant were retained and data on affected host, study area, study date, sample size, number of positives, affected age and sex whenever available, diagnosis method used, type of leishmaniasis studied as either cutaneous or visceral leishmaniasis, author's name, article title, and year of publication were recorded and extracted to a pre-prepared data extraction excel sheet.

### 2.3 Statistical analysis method

**Fig. 2:**
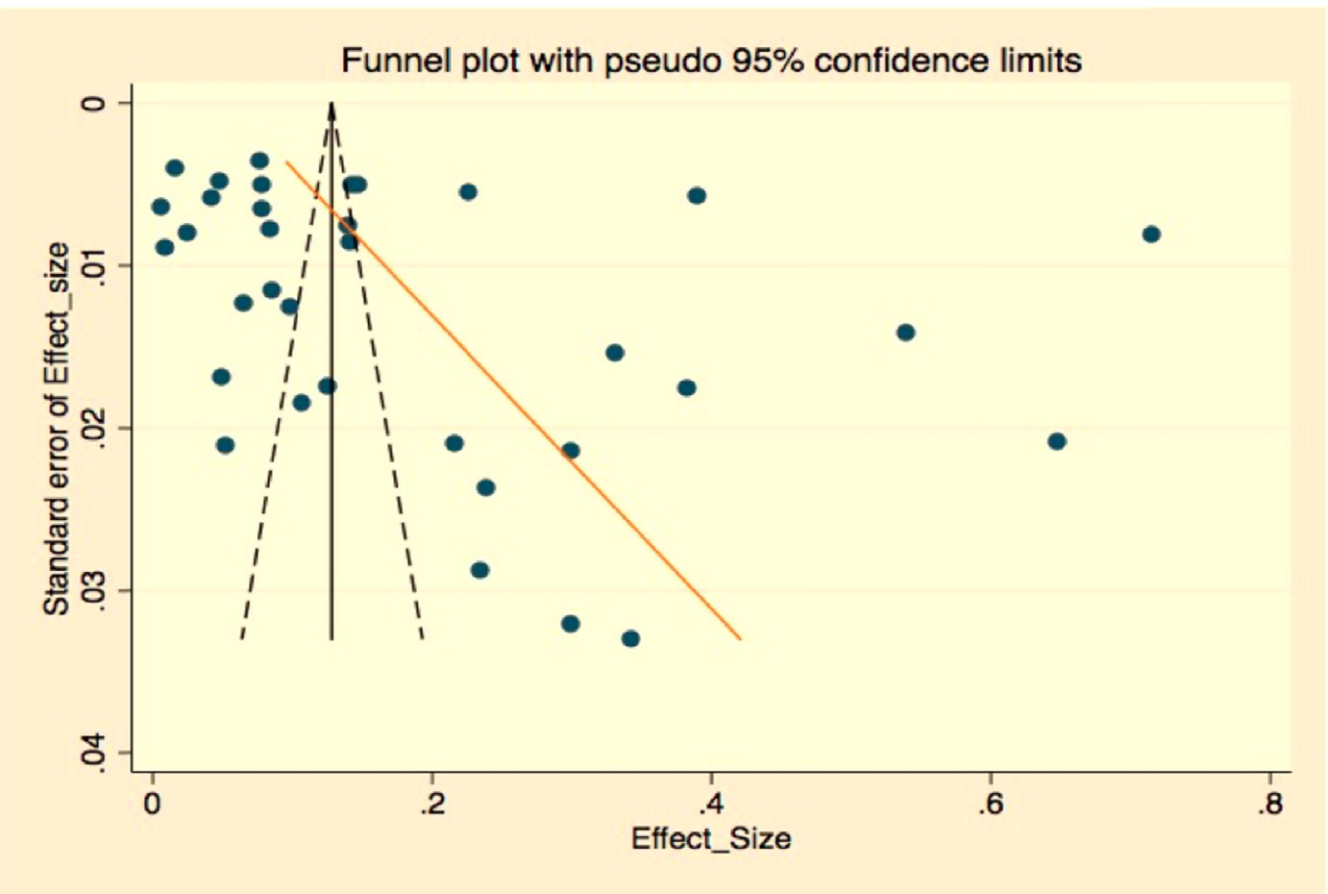
Funnel plot that assesses publication bias.

**Fig. 3:**
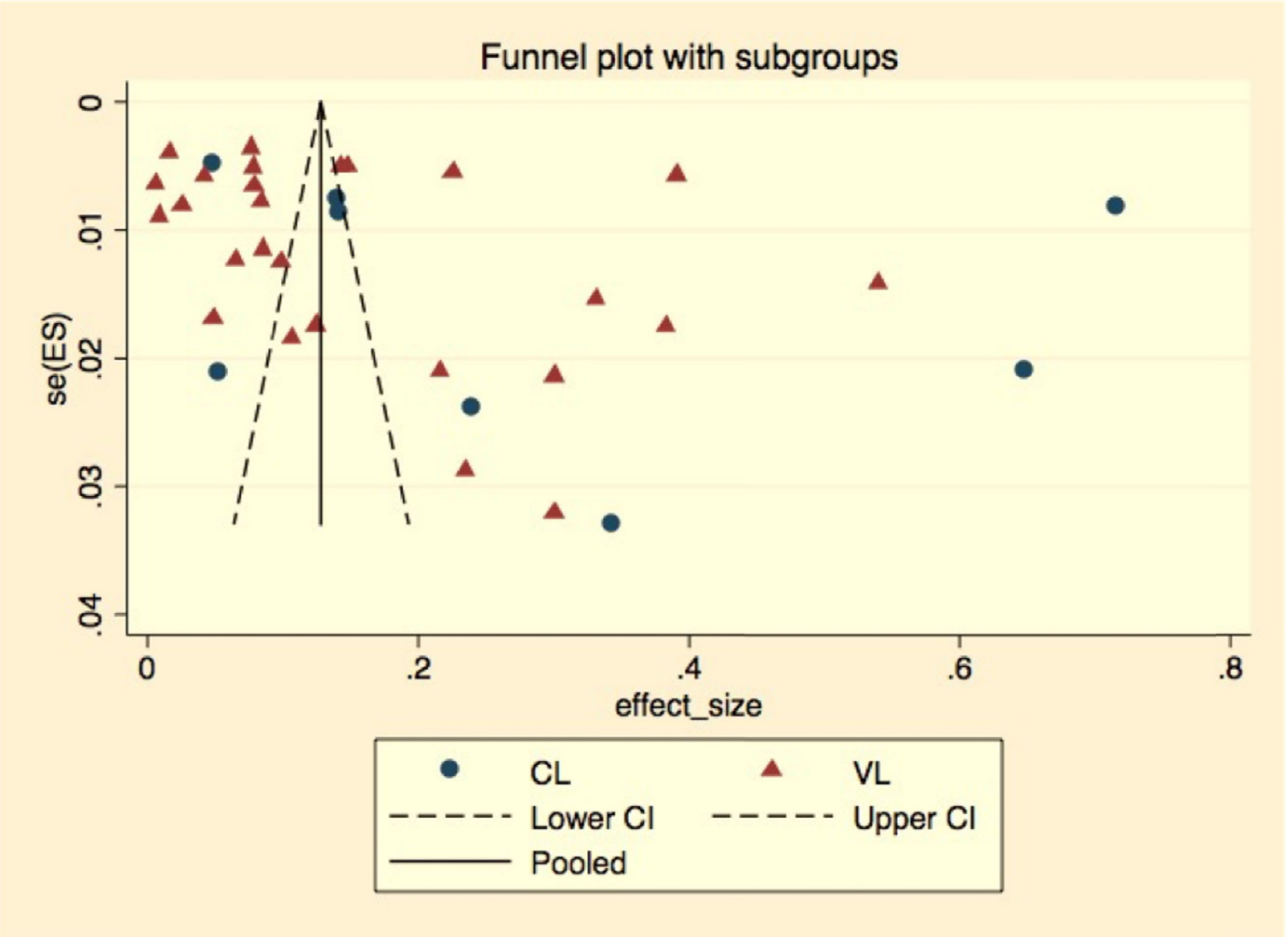
Funnel plot that assesses publication bias of both cutaneous and visceral Leishmaniasis.

Statistical analyses were done using the *metaprop_one* package of Stata 14 software. Descriptive statistics did a simple summary of reports with their apparent prevalence. Meta-analysis of prevalence data was analyzed and pooled the estimates and 95% confidence intervals. Because substantial heterogeneity was expected, random-effects meta-analysis was done with the estimate of heterogeneity being taken from an inverse-variance model (DerSimonian and Laird, 1986). Subgroup analysis was conducted and stratified the studies according to study location which was categorized as central, northern, southern, and western according to the reported studies location, type of leishmaniasis reported (visceral leishmanial or cutaneous leishmaniasis), affected hosts as either in human or animals to minimize heterogeneity of studies. Publication bias was assessed using the Begg & Eggers test, as well as visual inspection of the funnel plot (Figs. 2 and 3). Between study, heterogeneity was assessed using *I*^2^ and Cochran's Q method. *I*^2^ values of 25%, 50%, and 75% were considered low, moderate and high heterogeneity, respectively (Higgins and Thompson, 2002). Meta-regression was used to investigate factors potentially contributing to the between-study heterogeneity. Univariable analysis was done for variables; sample size as a continuous and categorized variable, study location, reported Leishmaniasis type, study year, affected host, and diagnosis method used.

## 3 Results

### 3.1 Descriptive results of eligible studies

From all screened studies, 30 were eligible for the final meta-analysis. Among these studies, 25 studies were on a human while the rest five studies were on animal reservoirs (cattle, sheep, goat, donkey, dog, rodents, and bats). Descriptive summary statistics were calculated to determine the total number of the population sampled and the range of prevalence estimates. Accordingly, the study characteristics were as follows. The total sample size throughout the study years were 52,706 animals and humans in which 11,504 were found positive for Leishmaniasis. The most extensive study regarding sample size employed 7,161 humans while the smallest study included 111 individuals. The overall apparent prevalence in all studies estimated was 19.1% in both human and animals. A detailed summary of the studies can be found in Table 1.

**Table 1:** Descriptive statistics of included studies in the final systematic review and meta-analysis.

S. N	SS	NP	AP	Diagnosis method	TL	Host affected	Study location	Study (references)
**1**	4757	680	14.29	Molecular diagnosis	VL	human	Northern Ethiopia	Abbasi et al. (2013)
**2**	5330	408	7.65	Parasitological examination	VL	human	Southern Ethiopia	Ali and Ashford (1994)
**3**	767	294	38.33	Serological examination	VL	human	Northern Ethiopia	Ali et al. (2005)
**4**	459	138	30.07	Molecular diagnosis	VL	human	Northern Ethiopia	Alvar et al. (2007)
**5**	1184	50	4.22	Serological examination	VL	human	Southern Ethiopia	Ayele and Ali (1984)
**6**	1672	132	7.89	Parasitological examination	VL	human	Central Ethiopia	Ayele (1988)
**7**	398	26	6.53	Serological examination	VL	human	Northern Ethiopia	Azene et al. (2017)
**8**	1651	234	14.17	Parasitological examination	CL	human	Central Ethiopia	Bekele et al. (2014)
**9**	2106	295	14.01	Molecular diagnosis	CL	human	Northern Ethiopia	Bsrat et al. (2015)
**10**	523	339	64.82	Serological examination	CL	human	Southern Ethiopia	Bugssa et al. (2014)
**11**	565	56	9.91	Serological examination	VL	human	Northern Ethiopia	Custodio et al. (2012)
**12**	1010	17	1.68	Serological examination	VL	human	Central Ethiopia	Degu (2006)
**13**	928	308	33.19	Serological examination	VL	human	Northern Ethiopia	Diro et al. (2015)
**14**	384	83	21.61	Serological examination	VL	human	Northern Ethiopia	Ferede et al. (2017)
**15**	4870	721	14.80	Serological examination	VL	human	Southern Ethiopia	Hailu et al. (1996)
**16**	2753	216	7.85	Serological examination	VL	human	Western Ethiopia	Hailu et al. (1996)
**17**	1232	665	53.98	Serological examination	VL	human	Southern Ethiopia	Hailu et al. (2009)
**18**	217	51	23.50	Serological examination	VL	Animals	Northern Ethiopia	Kalayou et al. (2011)
**19**	586	50	8.53	Molecular diagnosis	VL	Animals	Central Ethiopia	Kassahun et al. (2015a)
**20**	163	8	4.91	Molecular diagnosis	VL	Animals	Central Ethiopia	Kassahun et al. (2015b)
**21**	1280	108	8.44	Serological examination	VL	human	Northern Ethiopia	Kebede (2007)
**22**	203	61	30.05	Serological examination	VL	Animals	Northern Ethiopia	Kenubih et al. (2015)
**23**	207	71	34.30	Serological examination	CL	human	Central Ethiopia	Lemma et al. (1969)
**24**	322	77	23.91	Serological examination	CL	human	Northern Ethiopia	Lemma et al. (1969)
**25**	113	6	5.31	Serological examination	CL	human	Southern Ethiopia	Lemma et al. (1969)
**26**	359	45	12.53	Serological examination	VL	human	Northern Ethiopia	Lemma et al. (2015)
**27**	3022	2164	71.61	Parasitological examination	CL	human	Southern Ethiopia	Mengistu et al. (1992)
**28**	1907	92	4.82	Molecular diagnosis	CL	human	Southern Ethiopia	Negera et al. (2008)
**29**	155	1	0.9	Molecular diagnosis	VL	Animals	Northern Ethiopia	Rohousova et al. (2015)
**30**	111	1	0.65	Molecular diagnosis	VL	Animals	Northern Ethiopia	Rohousova et al. (2015)
**31**	280	30	10.71	Molecular diagnosis	VL	Animals	Northern Ethiopia	Rohousova et al. (2015)
**32**	5645	1276	22.60	Serological examination	VL	human	Northern Ethiopia	Shiferaw et al. (2016)
**33**	386	10	2.59	Serological examination	VL	human	Northern Ethiopia	Sordo et al. (2012)
**34**	7161	2801	39.11	Serological examination	VL	human	Northern Ethiopia	Wondimeneh et al. (2014)

**SS** sample size, **NP** number of positives, **AP** apparent prevalence, **TL** type of leishmaniasis.

**Fig. 4:**
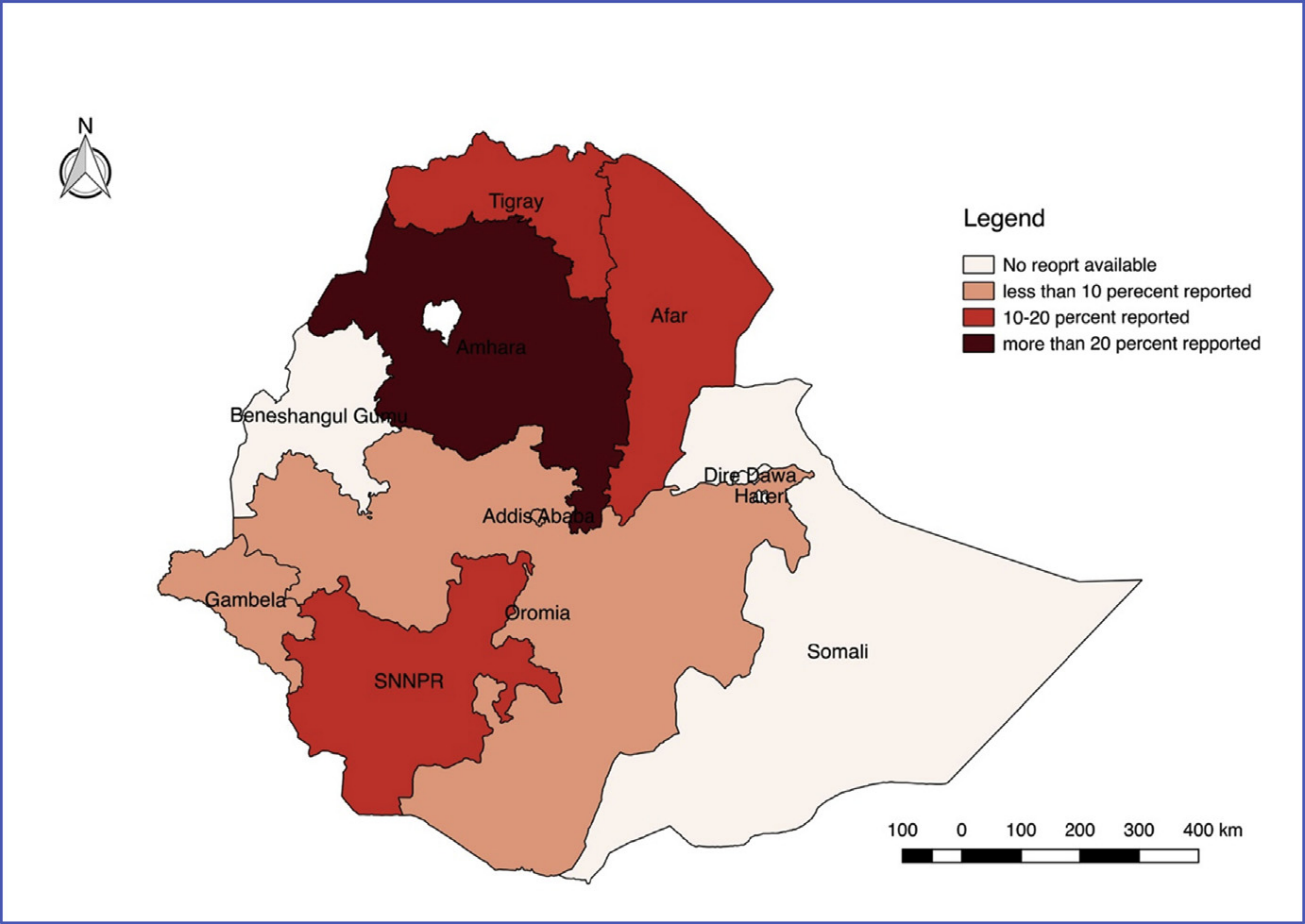
Spatial distribution of leishmaniasis in Ethiopia based on data extracted from eligible studies in the final systematic review and meta-analysis.

Moreover, leishmaniasis was highly prevalent in northern and southern parts of the country. Amhara region was the leading in the prevalence of leishmaniasis occurrence followed by Tigray and Southern Nations Nationalities Peoples Regional State (SNNPR). Based on the selected eligible studies, leishmaniasis was reported in 7 regions including Addis Ababa city administration. In the remaining three regions (Somali, Benshangul Gumuz, and Hareri) and Dire Dawa city administration, no eligible studies were found to be included in this study. Spatial distribution of prevalence of leishmaniasis according to study data is illustrated in Fig. 4.

### 3.2 Meta-analysis

**Fig. 5:**
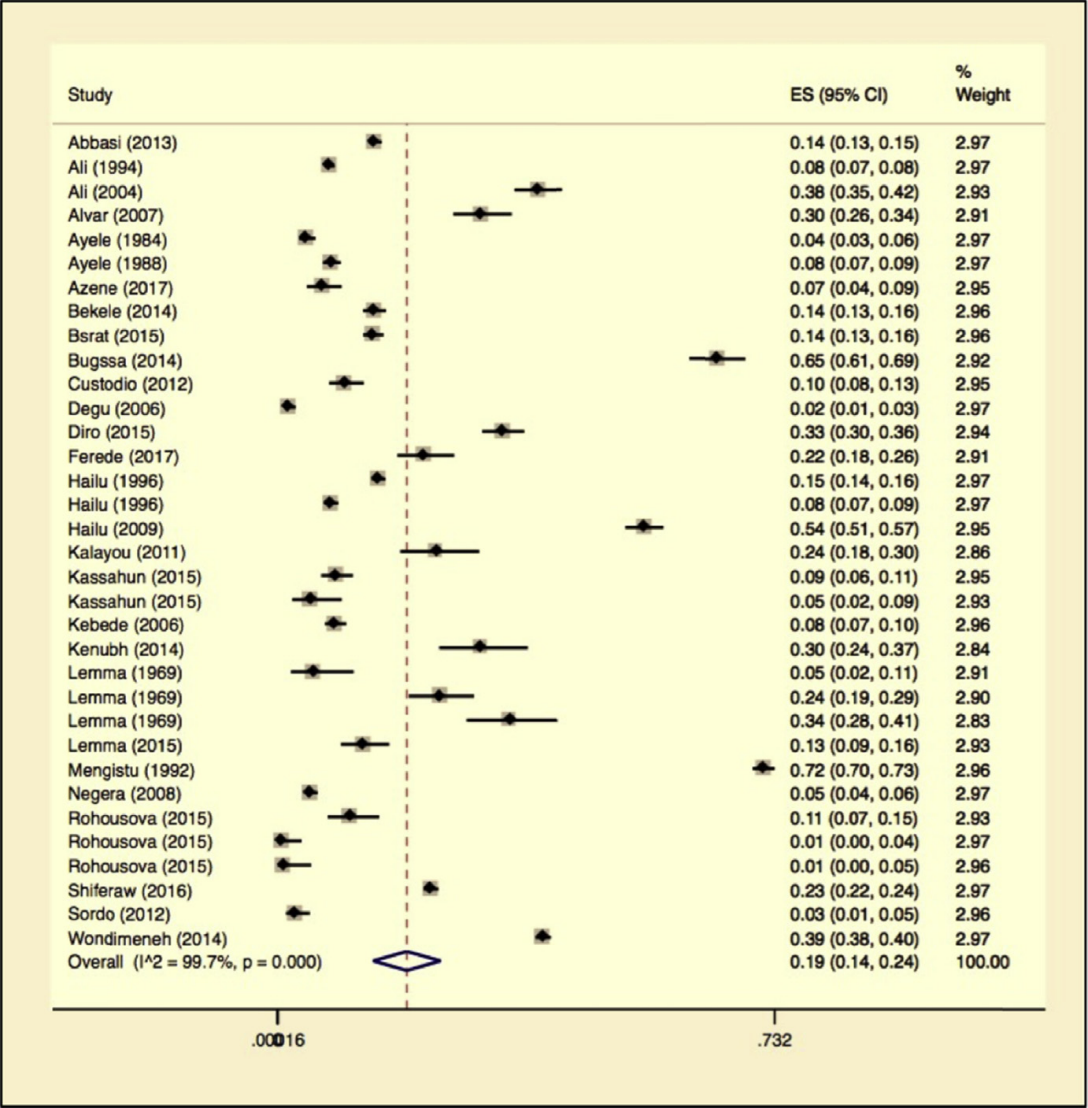
Forest plot on Leishmaniasis infection prevalence estimates in both animals and humans in Ethiopia.

Random-effects meta-analyses were carried out using the total sample size and number of positives (effect size, standard error of effect size) to estimate the prevalence of leishmaniasis at the country level. Between-study variability was high (τ^2^ = 0.02; heterogeneity I^2^ = 99.72% with Heterogeneity chi-square = 11985.41, a degree of freedom = 33 and P = 0.001). Individual study prevalence estimates ranged from 1% to 65% with the overall random pooled prevalence of 19% (95% CI 14%–24%). Studies weighted approximately equally with weights on individual studies ranging from 2.83% to 2.97 %. Fig. 5 presents the Forest plot derived from the meta-analysis.

### 3.3 Subgroup meta-analysis

**Fig. 6:**
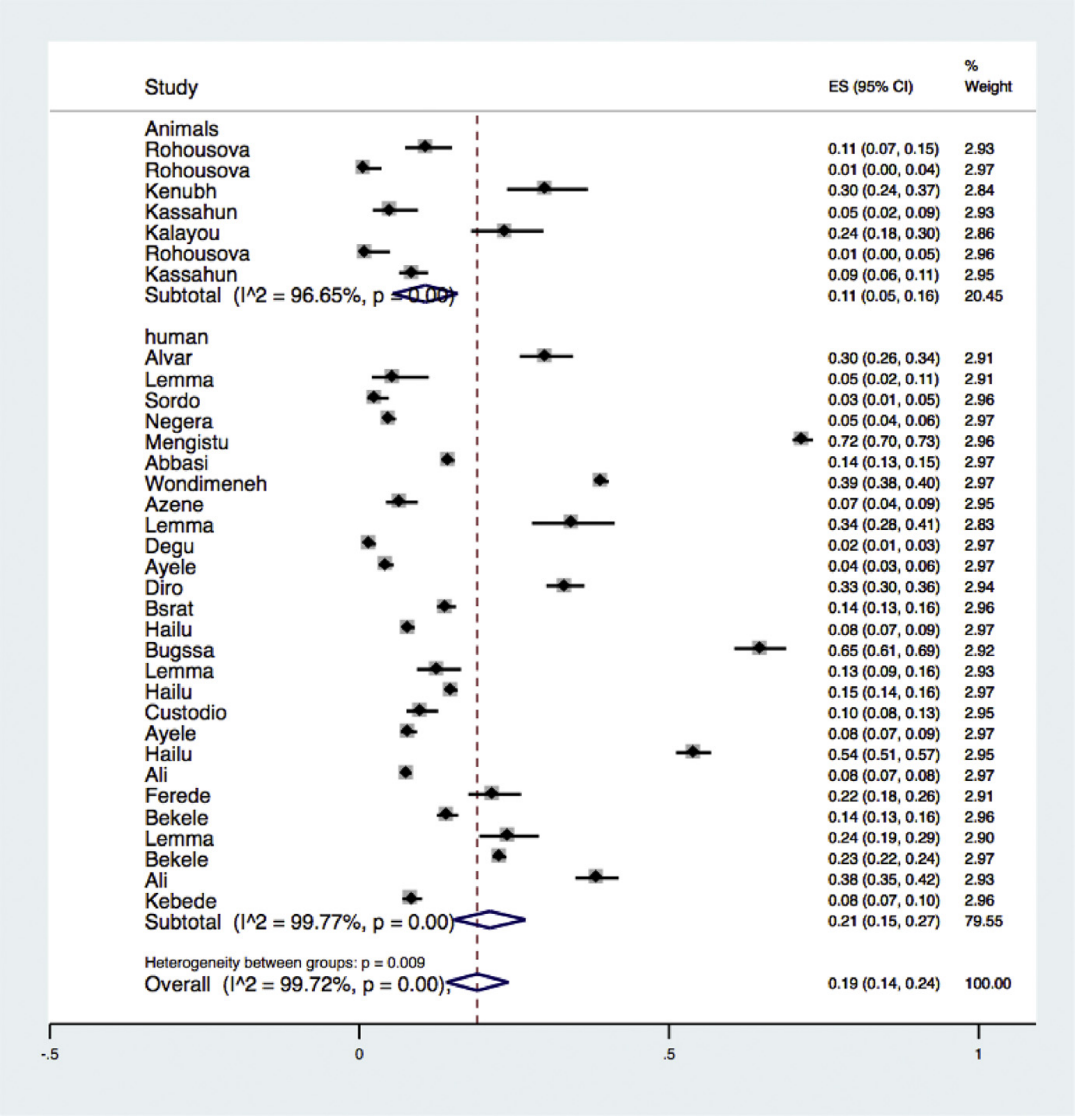
Forest plot on Leishmaniasis infection prevalence estimates by a subgroup analysis in animals and humans.

Subgroup analysis was done for selected groupings. Accordingly, the result of subgroup analysis by hosts affected (human or animals) indicated large variability in studies reporting prevalence of leishmaniasis in both human and animals (the Higgins I^2^ statistic = 99.8% with Heterogeneity chi-square = 11327.20, a degree of freedom = 26 and P = 0.001) in humans and (the Higgins I^2^ statistic = 96.65% with Heterogeneity chi-square = 179.13, a degree of freedom = 6 and P = 0.009) in animals. Sub-total random pooled prevalence of leishmaniasis was estimated at 11% (95% CI 5%–16%) in animals and 21% (95% CI 15%–27%) in human. The Forest plot is illustrated in Fig. 6 and the overall statistics in Table 2.

**Table 2:** Subgroup analysis for comparison of prevalence of leishmaniasis in human and animals.

Host affected	No. of studies	Prevalence 95% CI	I^2^%	Q	Heterogeneity test	
					DF	*P*
Human	27	11 (5–16)	99.77	11327.20	26	<0.001
Animals	7	21 (15–27)	96.65	179.13	6	<0.001
**Overall**	**34**	**19 (14–24)**	**99.72**	**11985.41**	**33**	<**0**.**001**

**Fig. 7:**
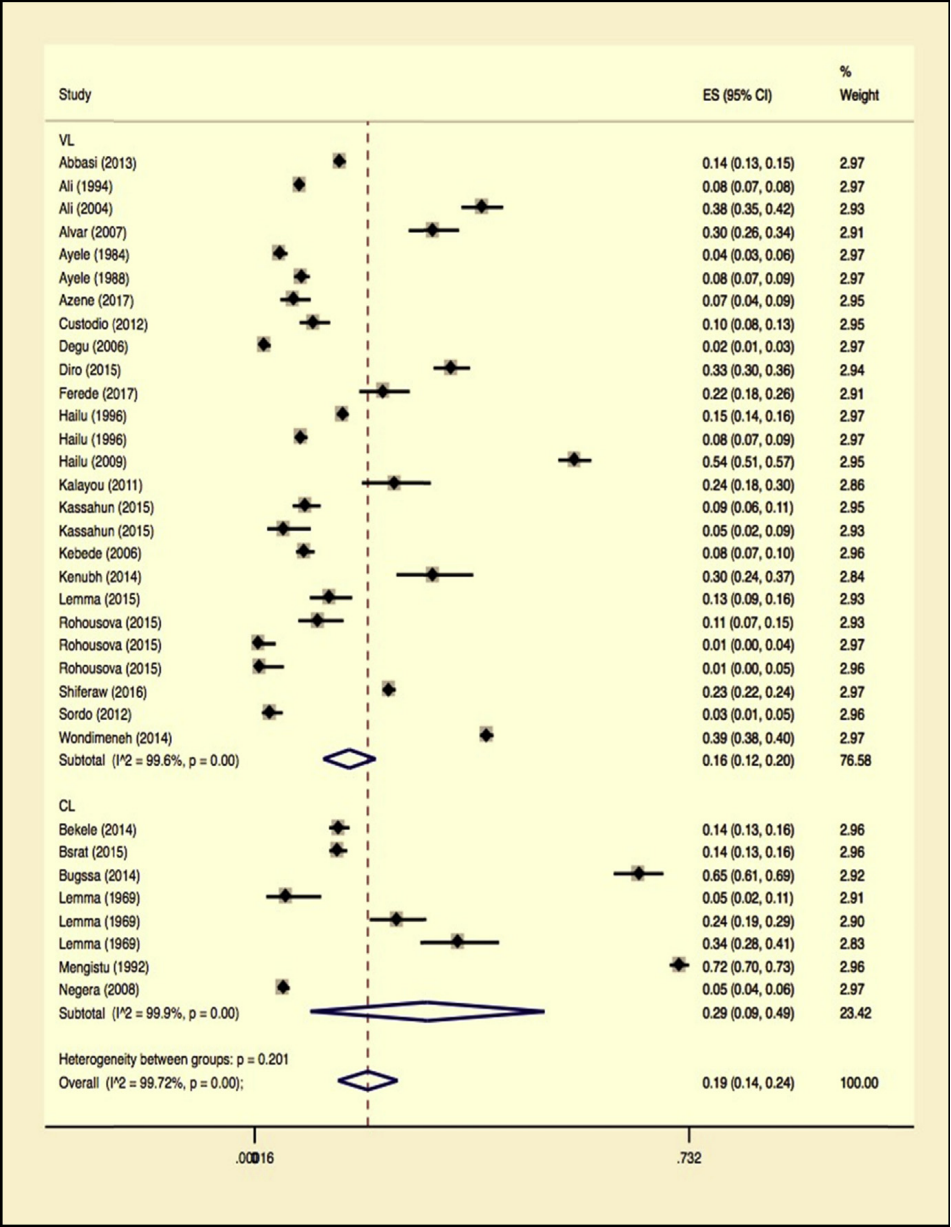
Forest plot on Leishmaniasis infection prevalence estimates subgroup analysis by type of leishmaniasis reported (cutaneous or visceral leishmaniasis).

The other subgroup analysis was conducted for the type of leishmaniasis involved. The result showed visceral Leishmaniasis was much higher reported than cutaneous leishmaniasis. Numerically, (the Higgins I^2^ statistic = 99.57% with Heterogeneity chi-square = 5773.21), a degree of freedom = 25 and P = 0.001 for visceral Leishmaniasis and (the Higgins I^2^ statistic = 99.87% with Heterogeneity chi-square 5553.01, a degree of freedom = 7 and P = 0.001) for cutaneous leishmaniasis. Sub-total, the random pooled prevalence of visceral Leishmaniasis, was estimated 16% (95% CI 12%–20%), while it was 19% (95% CI 14%–24%) for cutaneous Leishmaniasis. There was a significant difference between the two groups at p = 0.001. The Forest plot is illustrated in Fig. 7 and the overall statistics in Table 3.

**Table 3:** Subgroup analysis for comparison of prevalence by type of leishmaniasis (cutaneous and visceral leishmaniasis).

Type of leishmaniasis	No. of studies	Prevalence 95% CI	I^2^%	Q	Heterogeneity test	
					DF	*P*
**VL**	26	16 (12–20)	99.57	5773.21	25	<0.001
**CL**	8	19 (14–24)	99.87	5553.01	7	<0.001
**Overall**	**34**	**19 (14–24)**	**99.72**	**11985.41**	**33**	<**0**.**001**

**VL** visceral Leishmaniasis, **CL** cutaneous Leishmaniasis.

**Fig. 8:**
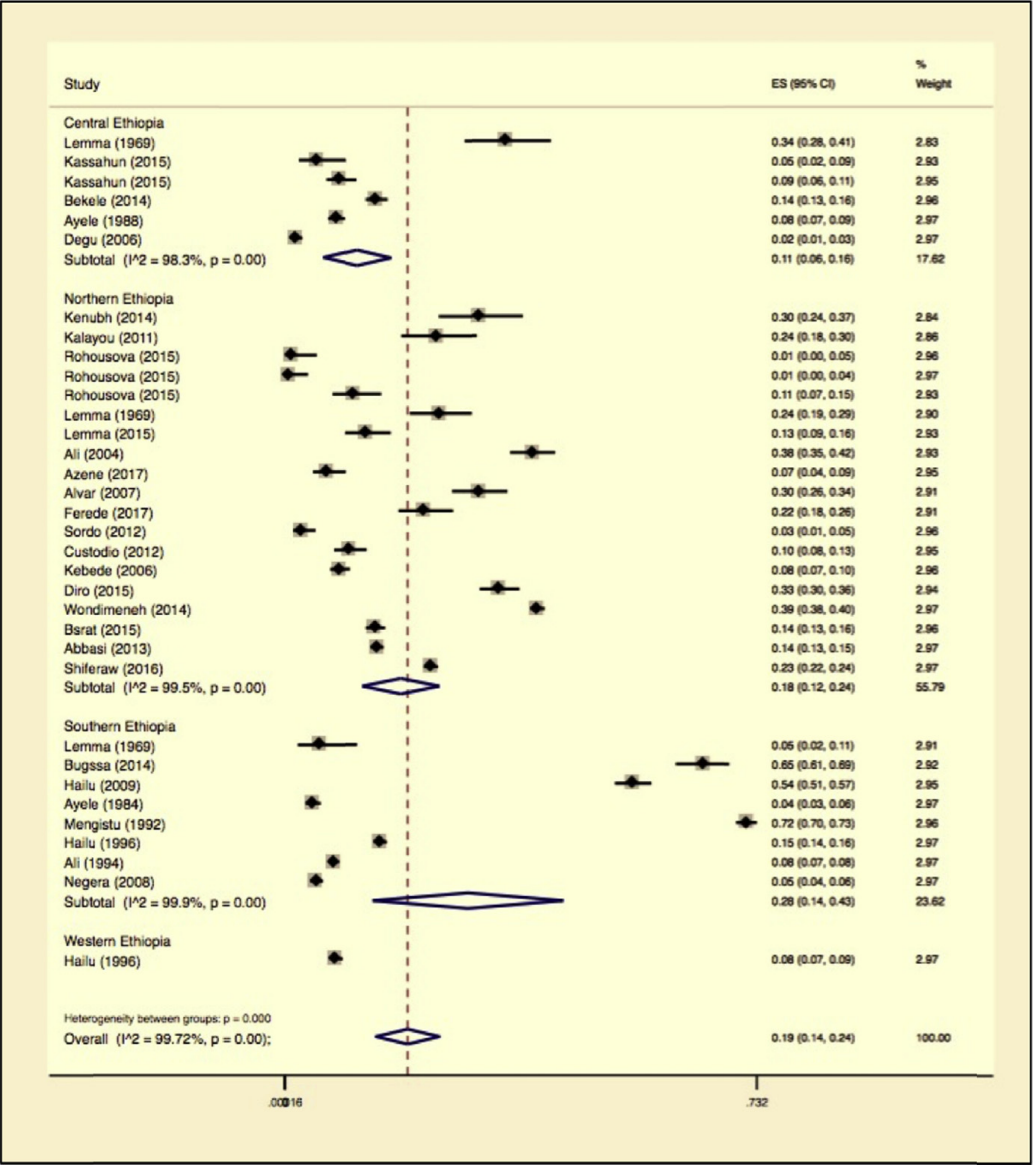
Forest plot on Leishmaniasis infection prevalence estimates subgroup analysis by study location.

Furthermore, subgroup analysis was computed for study location by classifying study sites as central, northern, southern, and western Ethiopia. There was a significant difference between study locations (p = 0.001). Subgroup analysis by location showed that much of the studies came from the northern part of the country followed by southern Ethiopia. We observed study variabilities in 3 locations while we omitted result of one location due to lack of enough number of studies (observations). The Higgins statistic were 99.48% with Heterogeneity chi-square = 3455.50, a degree of freedom = 18 and P = 0.001 for northern Ethiopia while the southern Ethiopia variability was with Higgins statistic of 99.9% with Heterogeneity chi-square = 7269.74, a degree of freedom = 7 and P = 0.001 for southern Ethiopia. The central Ethiopia reports had a variability of 98.26% with Heterogeneity chi-square = 287.26, a degree of freedom = 5 and P = 0.001. The subtotal pooled prevalence for central, northern and southern Ethiopia was 11% (95% CI 6%–16%), 18% (95% CI 12%–24%) and 28 % (95% CI 14%–43%) respectively. The Forest plot is illustrated in Fig. 8 and the overall statistics in Table 4.

**Table 4:** Subgroup analysis for comparison of prevalence of leishmaniasis in different geographical locations across Ethiopia.

Region	No. of studies	Prevalence 95% CI	I^2^%	Q	Heterogeneity test	
					DF	*P*
Central Ethiopia	6	11 (6–16)	98.26	287.26	5	<0.001
Northern Ethiopia	19	18 (12–24)	99.48	3455.5	18	<0.001
Southern Ethiopia	8	28 (14–43)	99.90	7269.74	7	<0.001
Western Ethiopia	1	8 (7–9)	-	-	-	-
**Overall**	**34**	**19 (14–24)**	**99.72**	**11985.41**	**33**	<**0**.**001**

### 3.4 Meta-regression

**Fig. 9:**
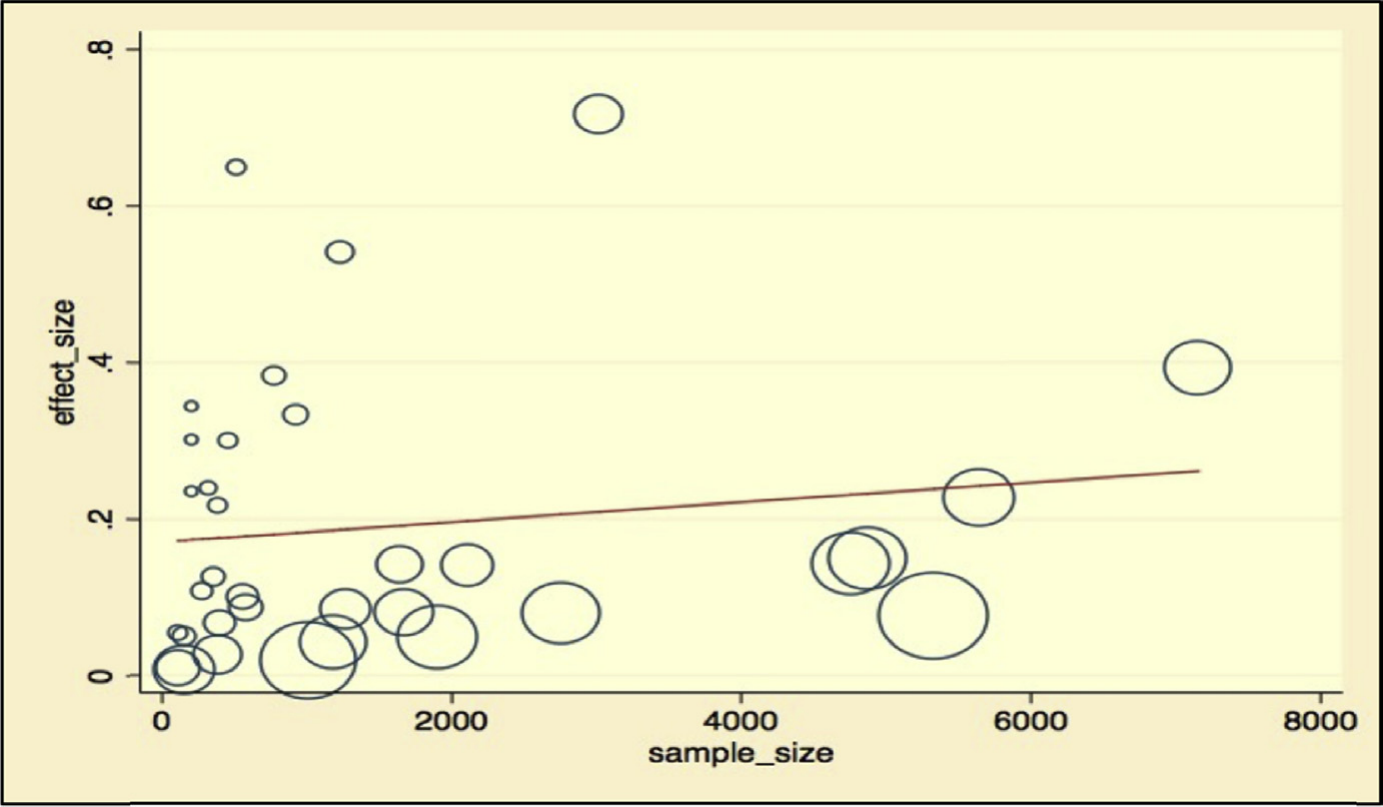
Meta-regression plot of sample size versus prevalence of leishmaniasis in Ethiopia.

**Fig. 10:**
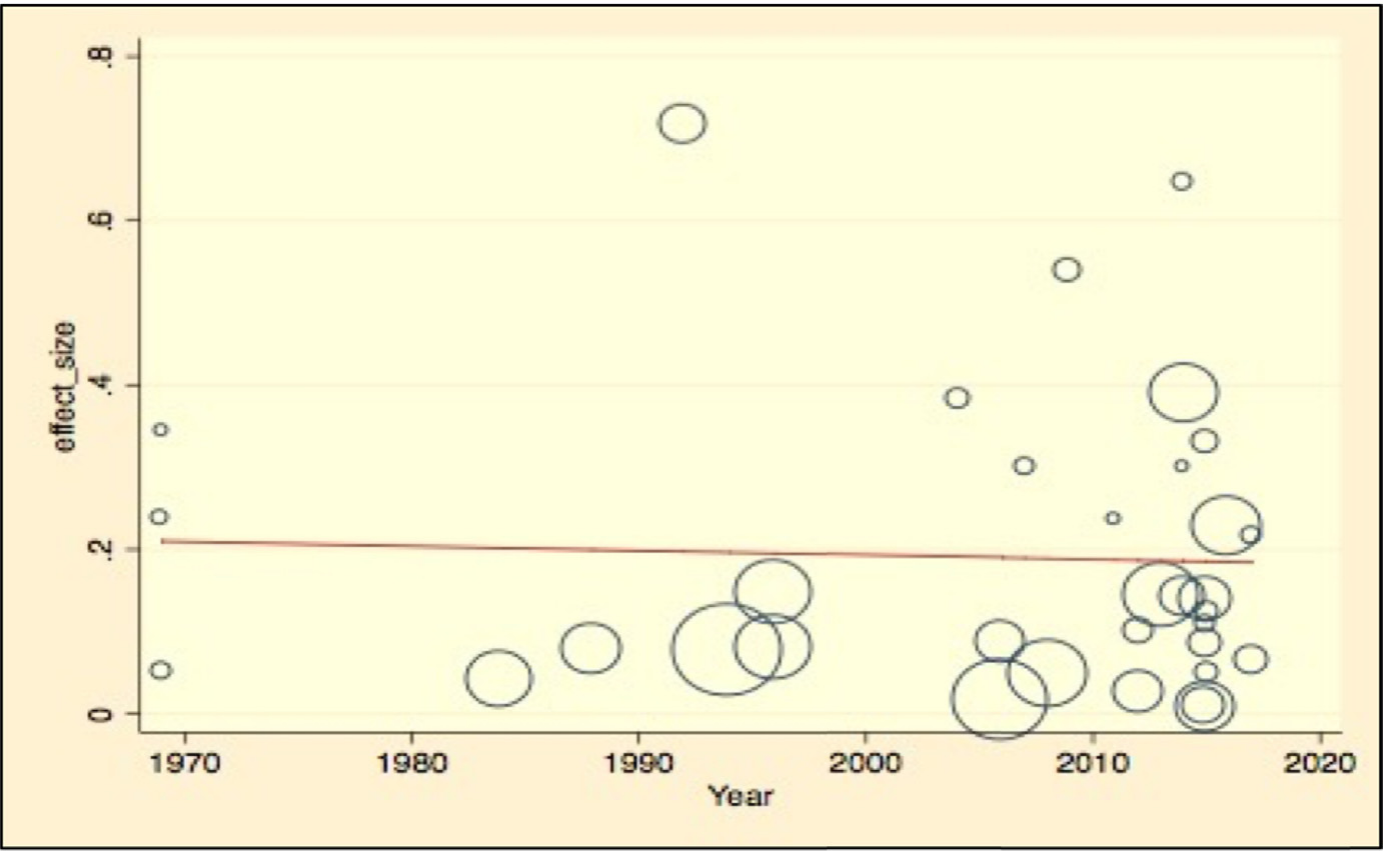
Meta-regression plot of study year versus prevalence of leishmaniasis in Ethiopia.

**Fig. 11:**
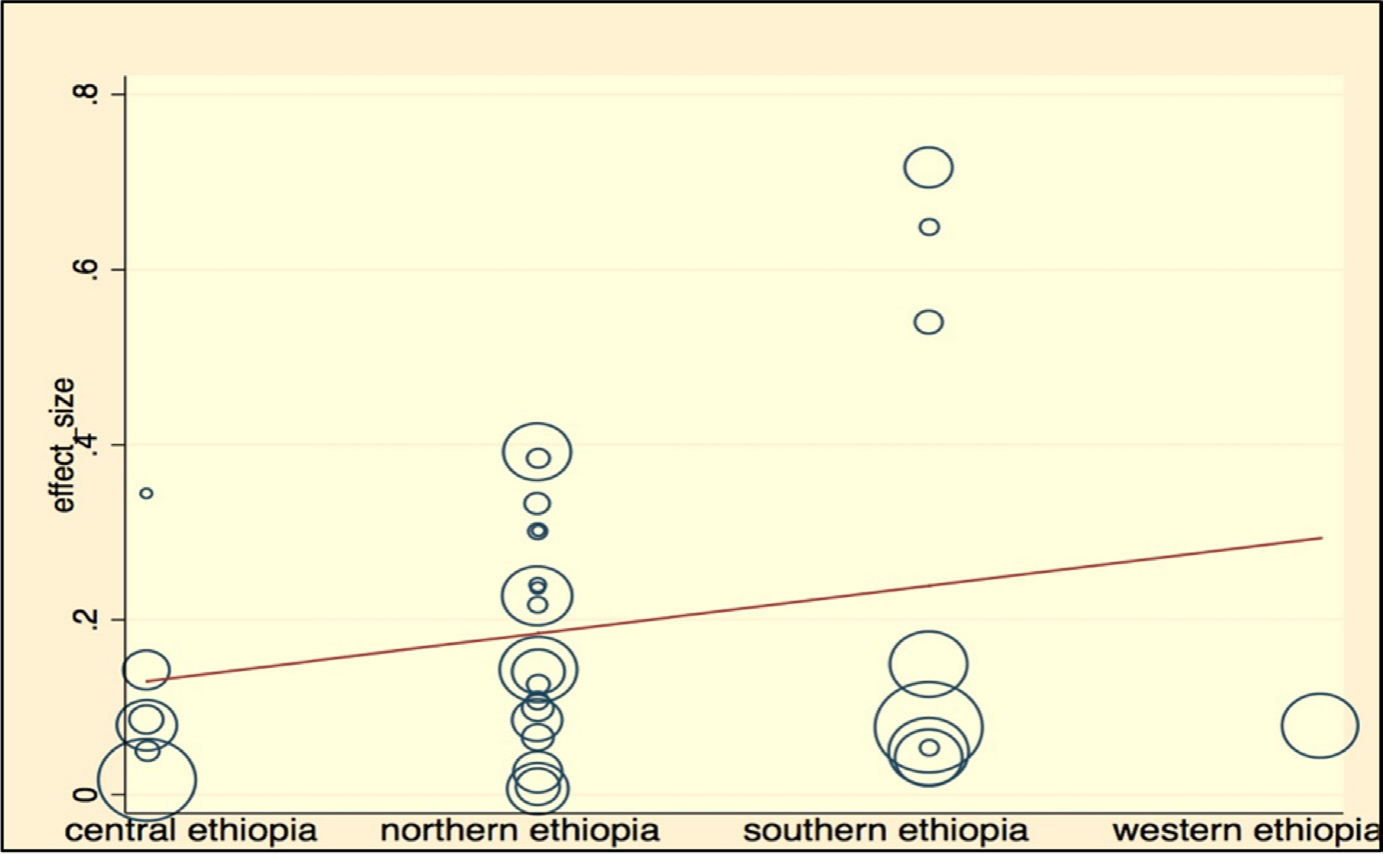
Meta-regression plot of study location versus prevalence of leishmaniasis in Ethiopia.

Meta-regression analysis was done for each variable included in the study individually. The variables included were sample size as a continuous variable and categorized variable, study location, leishmaniasis type reported, study year, affected host, and diagnosis method used (by categorizing them as serological diagnosis, parasitological examination, and molecular diagnosis). Continuous variables were subjected to assessment to see a linear relationship with the independent effect size. Those variables with p-values <0.25 were used in the multivariable meta-regression analysis. Independent variables like categorized sample size, hosts, diagnosis method used, and study location had reasonably significant value and retained in the final multivariate analysis. Most of the variables were not significantly associated with the prevalence of Leishmaniasis in the final multivariate meta-regression. However, the diagnosis method had a contribution to the heterogeneity of studies. Molecular diagnosis has significantly lower prevalence than microscopic examination of parasites with a coefficient of −0.32, and a p-value of 0.024, with 95% CI (−0.6–0.05). The results of the final meta-regression analysis have been summarized in Table 5, and meta-regression graphs are illustrated in Figs. 9, 10, and 11.

**Table 5:** Final multivariable meta regression model.

Variables	Coefficient	P-value	95% CI
**Diagnosis method**			
Parasitological microscopic examination	Reference		
Serological diagnosis method	−0.14	0.2	−0.39–0 .11
Molecular diagnosis	−0.32	0.024	−0.60–0.05
**Sample size category**			
Sample size less than 350	Reference		
Sample size between 351- 565	0.19	0.122	−0.06–0.44
Sample size between 566–1000	0.12	0.403	−0.17–0.41
Sample size more than 1000	0.2	0.169	−0.08–0.45
**Type of leishmaniasis**			
Cutaneous leishmaniasis	Reference		
Visceral leishmaniasis	−0.16	0.118	−0.37–0.045
**Affected hosts**			
Animals	Reference		
Humans	−0.17	0.271	−0.47–0.12
**Study location**			
Central Ethiopia	Reference		
Northern Ethiopia	0.12	0.239	−0.08–0.31
Southern Ethiopia	0.11	0.302	−0.11–0.33
Western Ethiopia	0.03	0.893	−0.42–0.48
**Constant**	**0.3180838**	**0.031**	.**031**–**0**.**60**

### 3.5 Publication bias and small study effect assessment

Publication bias and small study effects were assessed by funnel observation and Egger's test for small-study effects. The result of effect estimates against its standard error showed that there was no publication bias with a p-value of 0.084 (Table 6 and Fig. 2 for funnel plot assessment).

**Table 6:** Eggers test for publication bias assessment.

Standard Effect	Coefficient	t-value	p-value	95% CI
**Slope**	0.055	1.16	0.256	−0.042–0.15
**Bias**	11.08	1.78	0.084	−1.57–23.74

## 4 Discussion

To the best of our knowledge, this is the first systematic review and meta-analyses on the prevalence of Leishmaniasis in Ethiopia. The results presented in this report were from the analysis of data obtained through a systematic review of scientific publications of the prevalence of at country level between the years of 1969–2017. Literature was heterogeneous, had inappropriate study designs, unrepresentative sample size, diagnostic methods. This diversity, together with the lack of data on other required variables, reduced the datasets substantially. The final quantitative and meta-analysis of the prevalence were done only on 25 articles in human and five articles on animals.

The random effect meta-analysis result showed a high variability with Higgin's I^2^ which indicate that the variability between studies was not by chance alone. Because of the considerable variability between studies, the random effects meta-analysis weight of studies was nearly equal. Diagnosis method was a highly significant predictor of the prevalence of leishmaniasis indicating that this variable explains a substantial portion of the variability between studies. However, other variables retained in the final meta-regression seems statistically insignificant in explaining the study variability.

This review demonstrated that there is still a significantly higher prevalence of leishmaniasis in Ethiopia. A pooled prevalence of 19% at the country level needs critical attention from responsible bodies of the country. The disease is a neglected tropical disease with devastating impact in the poor third world countries (Ngure et al., 2009). The presence of immune compromising conditions like HIV/AIDS and malnutrition in these countries make its impact severe (Alvar et al., 2008, 2006). The epidemiology of the diseases is mostly determined by many factors including but not limited to; suitable environment for vectors, the presence of reservoir, intense agricultural practices, and migration. All those factors are met in northern and southern parts of Ethiopia (Ali et al., 2016; Gadisa et al., 2015; Ngure et al., 2009), which is why most of the studies reported from these localities indicating the importance of the disease in Humera and Metema of Northern part and the Rift valley areas of southern parts of the country. The presence of higher incidence of HIV/AIDS in these Humera and Metema districts can be another reason for the increased prevalence of leishmaniasis (Alemayehu et al., 2017; Mengistu and Ayele, 2007).

According to this systematic review and meta-analyses (SR&MA), the most dominant type of Leishmaniasis in Ethiopia is visceral Leishmaniasis. The disease has been reported in many parts of the country. However, it was found to be much more devastating in northern Ethiopia, particularly Libokemkem, Metema and Humera districts (Ferede et al., 2017; Mengesha et al., 2014; Welay et al., 2016). Moreover, cutaneous leishmaniasis is also a well-established in the country. Mainly it was indicated in Kutaber districts of northern Ethiopia, and most importantly, it is highly prevalent in rift ally area of the southern part of the country (Lemma et al., 1969; Negera et al., 2008).

In the literature search stage of this SR&MA, we noted that finding an article reporting animal reservoirs was difficult. Only a few (5) publications made it to the final reporting of this SR&MA. A pooled prevalence of 11% leishmaniasis in animals may not be the exact reflection of the current status in Ethiopia. This review is a timely reminder of the need for more studies on animal reservoirs. Studies should be routinely implemented in endemic foci and other parts of the country. In relative terms, one can say that human leishmaniasis has been well studied in the country. However, this does not mean that the current studies of human leishmaniosis can explain the exact status of the diseases across Ethiopia. Reporting on risk factors as sex and age affected, the presence dogs in the household, species of *Leishmania* involved and many more other risk factors reviewing were not possible in this review because of an incomplete report by studies included. Epidemiological investigations to assess the risk factors for the occurrence of Leishmaniasis need to be addressed. However, even with those limitations, this review noted that the importance of the diseases and could be a call for nationally coordinated control programs.

Leishmaniasis was studied in Ethiopia for many years. In this report, we were able to employ studies that were done back in 1969. From that time to date many studies have been done with different objectives. Studies that can meet our objective were done variably throughout these years. This study indicated that there is an increase in number and quality of studies regarding diagnostic approach in recent years. Advances in the diagnostic approaches may be due to the advancement in researches and innovations of modern technologies, and relatively a better attention has been given to neglected tropical diseases recently.

**In conclusion,** this systematic review and meta-analysis showed that leishmaniasis infection is still endemic and highly prevalent in Ethiopia. Problems in studies like poor design, lack of internal validity and, many more reasons which result in reporting this meta-analysis in fewer reports than anticipated. High variability between studies may influence to estimate pooled prevalence at the national level. However, the report indicated that nationally coordinated prevention, control, and eradication program should be in place to reduce public health and socio-economic impact of the disease. The available studies in animal reservoirs were fewer than human leishmaniasis. Researches need to be updated on animal reservoirs to plan an efficient control strategy.

## Declarations

### Author contribution statement

Ayalew Assefa: Conceived and designed the analysis; Analyzed and interpreted the data; Contributed analysis tools or data; Wrote the paper.

### Funding statement

This research did not receive any specific grant from funding agencies in the public, commercial, or not-for-profit sectors.

### Competing interest statement

The authors declare no conflict of interest.

### Additional information

No additional information is available for this paper.
